# The Beddoe Memorial Lecture: Anthropology: Old and New

**Published:** 1930

**Authors:** Arthur Keith

**Affiliations:** Conservator of Museum, Royal College of Surgeons of England


### THE BEDDOE MEMORIAL LECTURE:

DELIVERED AT BRISTOL ON FRIDAY, 5TH SEPTEMBER, 1930, UNDER THE AUSPICES OF THE BRITISH ASSOCIATION FOR THE ADVANCEMENT OF SCIENCE,

BY

SIR ARTHUR KEITH, F.R.S.,
*Conservator of Museum, Royal College of Surgeons of England,*

ON

### ANTHROPOLOGY: OLD AND NEW,

BEING AN ACCOUNT OF WHAT DR. JOHN BEDDOE, A PHYSICIAN OF BRISTOL, DID FOR BRITISH ANTHROPOLOGY, TOGETHER WITH A PROPOSAL FOR THE FOUNDATION OF A CHAIR OF ANTHROPOLOGY IN THE UNIVERSITY OF BRISTOL.

---

WHEN the British Association met in Bristol in 1875, Dr. John Beddoe, one of its leading physicians, was invited to be President of its Anthropological Section. He declined the honour, for a reason which he divulged in his Autobiography,[1] published in 1910—just a year before his death at the age of 85. "I could not afford," he says, "to let my scientific reputation injure my medical practice." The incident deserves our consideration for several reasons. When we look back now to the times in which Dr. Beddoe lived and worked, and note the doings of the men who were searching into the beginnings of the British people, we see that we owe more to him than to any other anthropologist of the Victorian epoch. It was he who laid our present knowledge upon a sure basis of fact. If he had been a young man just beginning the practice of his profession when the British Association met here fifty-five years ago we could have understood his reluctance. But he was then 49, serving the Infirmary as physician, and after eighteen years of practice had won for himself a leading place amongst the medical practitioners of Bristol. Yet he turned the proffered honour aside, fearing that if he appeared before his fellow-citizens in the rôle of anthropologist they might lose faith in him as a physician.

Whether the public is right in believing that a professional man cannot serve two mistresses at once I shall not stay now to discuss. The prejudice is old, and we have simply to note here that Dr. Beddoe rightly or wrongly bowed to it. Anthropology had become his favourite subject, "as it almost always becomes with those who once taste its delights and interests." He had set out in his youth to find an answer to an age-old query : Of what race or races are we British ? The more he searched the quicker did his interest in the problem become. By 1875 it had become the dominant objective of his life. To continue his anthropological campaigns in holiday times and in leisure hours he had to make sure that his practice would provide the necessary ways and means. If his practice failed his objective had to be abandoned. The incident throws light on the manner in which anthropology was cultivated in our country in the nineteenth century, and also tells us much about the nature of the man whom I regard as the founder of modern physical anthropology. Dr. John Beddoe had to be an anthropologist by stealth.

*England's Brain Belt.*

Let us examine very briefly the circumstances which turned Dr. Beddoe's mind towards the study of anthropology. We have to note the place of his birth; he was born in Galton's "brain belt"—which, passing from Liverpool to Swansea, includes the English counties bordering on Wales. I apologize for the fact that Sir Francis Galton,[2] in continuing his belt of genius southwards, carried it straight across the channel to fall on the borderlands of Cornwall and Devon—thus omitting Bristol and Somerset. Nevertheless, Bristol is indebted to this belt for many of her most distinguished citizens. It gave her Dr. James Cowles Prichard, who made England famous in the annals of anthropology during the first half of the nineteenth century. He was born at Ross-on-Wye, Hereford, in 1786; he practised as a physician in Bristol for thirty-eight years—from 1810 until his death in 1848. Dr. John Beddoe, who was destined to continue the reputation which Prichard had established for Bristol as a centre for anthropological inquiry, was born in 1826, a little farther along the belt—at Bewdley on the Severn—just within the western confines of Worcestershire—a town made famous in more recent days by its association with Mr. Baldwin. Still farther north in the belt—at Welshpool—was born in 1838 another eminent anthropologist—the late Sir William Boyd Dawkins. This belt, which has given England so many of her distinguished men of science, coincides with an old ethnological frontier, across which Saxon and Celt have freely exchanged their heritage of blood. The two Bristol anthropologists, Prichard and Beddoe, were Welsh in name. Dr. Beddoe estimated that the brain belt we have been considering was at least one-third Welsh in its racial constitution, and was of opinion it was in the process of becoming not less but more Welsh.

*How Beddoe became an Anthropologist.*

If we hesitate to attribute the natural endowments of our Bristol anthropologists to the fortune of being born on an ethnological frontier, we are on more certain grounds when we conclude that it was their place of birth which turned their minds to the problems of race. While still living in his father's comfortable and well-appointed home at Bewdley young Beddoe listened to discussions concerning the Celts and found divergence of opinion as to what sort of people they were, especially as regards their colouring. Some affirmed they were a fair people, others that they were dark. Even when he consulted authorities he found they depended not on exact figures but on general impressions. No one, he discovered, had ever made a census of the colouring of the people called Celts, or of any other race of mankind. He determined to set to work and investigate the colouring of British races by exact methods. He had made his first attempts in 1846, the year in which he was articled to a firm of solicitors, being then twenty years of age. He entered a virgin field of investigation ; it was necessary for him to invent methods of making records of colour of hair, of eyes, and of complexion ; his methods had to be such as permitted him to apply them as he passed along a village street or threaded his way among the throng in a market-place. After spending a year in a lawyer's office he passed on to the study of medicine in University College, London (1847–52), and it was in his student days that he first visited Ireland, Wales, the Isle of Man and the Highlands of Scotland, and France, applying and perfecting his methods of observing and of recording the colouring and other racial characteristics of the people he met with. In 1852 he settled down to his investigation in earnest. He went then to Edinburgh, and having passed his first medical examination there set out for the Orkney and Shetland Islands. After assisting a party of archæologists to excavate some ancient burials in Orkney, he applied his matured methods to the natives of these islands—people reputed to be of pure Norse descent. Then, having taken his medical degree in Edinburgh (1853), he made a prolonged tour round Scotland, tarrying in the valley of the Tweed to win the love of Agnes Montgomerie, who five years later became his wife and made him for ever afterwards a Scotophile Englishman. Otherwise his investigations were utterly devoid of racial prejudice.

*A New Departure in Anthropology.*

The result of this tour was the publication of his first important paper: "A contribution to the Ethnology of Scotland." Its appearance made no stir in the anthropological world; no one perceived that this young medico had made a new departure in our search into the history and relationships of the diverse peoples of Europe.

*As Surgeon and Anthropologist in the Crimean War.*

After his anthropological investigations of the Scottish people and a year spent in the Royal Infirmary,

Edinburgh, came the Crimean War and a dearth of medical men. Beddoe volunteered, and thus obtained opportunities, in the intervals of duty, of making anthropological raids into that hub of ancient humanity —the southern shores of the Bosphorus. He made acquaintance with the racial types of the East—Turks, Greeks, Bulgars, Armenians, Jews and Gentiles, and thus carried back from the war not only an enhanced medical experience but systematic records of the people he met with, records which in due time he could compare with others made in the British Isles. Circumstances provided young Beddoe with anthropological opportunities, but in all Europe there was no one but him who knew how to use them.

*Begins a Survey of Western Europe.*

No sooner was he home than he made up his mind to spend the winter of 1856–57 in medical study abroad, selecting Vienna as his centre. Mark the circuitous route he took to reach that city. It took him along the countries lying on the western shore of the North Sea — through Holland, Friesland, Westphalia, Hanover—to study the cousins of the English Saxons. He found himself in the Thuringian Hills, ascended the Elbe, loitered in Prague, studied the Czechs and the Slovaks, and ultimately reached Vienna, with sheafs of records—all duly documented. He attended the hospitals and spent his spare time unravelling the racial constituents of the population of Vienna. When the spring came he turned southwards, notebook in hand, passing through Styria, Croatia, until Italy was reached. Here he halted at over a score of centres to make records of the Italian people, and then made his way homeward through France. When in 1857 he settled down to practise in Bristol there was no one in Europe who had his exact knowledge of western peoples. In Bristol he purposely hid his anthropological light under a bushel; he did not want the people of Bristol to know he was an anthropologist, he desired to pass for what he was—a skilled physician deserving the confidence of the sick.

*A New Kind of Recreation—The Home of the Celt.*

There was not a single year between 1857, when he set up in practice, until 1891, when he retired to seek seclusion at Bradford-on-Avon, that Dr. Beddoe did not carry out an anthropological raid of some kind—in Ireland, Wales, Scotland, and England—every county of which he ultimately visited. From these raids he brought back records of hair colour, eye colour, and complexion of the people he met with, and charted them out on his maps. Often observations were made on size of head and form of face as well as on colouring. One can understand the attractions which Brittany, Normandy and Belgium had for him. He had found the home of the Saxons—but where did the British Celts come from? Belgium, he believed, provided him with what he was in search of—the home of the English Celts. In that country he found a sharply-demarcated racial frontier; the fair Flemings lay to the west of it, the darker Walloons to the east. The Walloons he regarded as descendants of the continental stock which had given Britain her Celts. He looked upon the people of Cornwall as cousins of the Walloons. He came across the same stock in south-west Scotland—in Upper Galloway and Strathclyde. The tall men, with brown hair and light grey eyes, so common in Ireland, he also regarded as British Walloons; he observed the same type amongst the people of Wales. Dr. Beddoe's theory that the Walloons of Belgium and the Celts of Britain had a common origin met with little credence in his day, nor in ours, and yet it is more than probable that the continental stock which gave Belgium her Walloons did provide ancient Britain with a considerable proportion of her population.

*The Effects of City Life on the People of England.*

During his busy years in Bristol Dr. Beddoe never ceased collecting such facts as could throw light on the racial constitution of the British people. There was, however, another and very important side to his inquiries. There were many in Dr. Beddoe's day who feared that industrialism would ultimately undermine the health and strength of the people of England, but he was one of the few to pass from speculation to actual inquiry. If he could only find out what was happening to the people of Bristol, he would then know how it fared with those masses of our racial stock which have become segregated in other great cities of England. So he set to measure—measure height, weight, size of head, colour of hair and of eyes—not only of every stratum of the population of Bristol, but of the open-living inhabitants of surrounding counties—from which Bristol has drawn her recruits. He took heights and weights of professional men, merchants, shipwrights, wheelwrights, corn porters, hauliers—indeed of all the representative trades of Bristol. The tallest were the "clickers" (5 ft. 9·3 in.), the shortest the tailors (5 ft. 5·2 in.). He found that the mean stature of the Bristolians measured by him was 5 ft. 6·5 in.; the North Somerset country-folk were just a little taller—5 ft. 6·9 in. He measured the colour of hair and eyes in 6,750 inhabitants of the city, representing all elements of the population. On his exact scale of reckoning the colouring of the Bristolians stood at 28·8—almost the same as that of Londoners. They were, however, eighteen points darker than the farming people of East Anglia, and thirty points more fair than the average crowd of South Wales. In point of colouring the people of Bristol may be said to be two-thirds Saxon and one-third Welsh. The country people of South Gloucestershire and North Somerset he found to be darker than the people of Bristol—their index being 38·6—midway between the East Anglian country-folk and the people of South Wales. They were half Saxon, half Welsh!

*The Census taken by Dr. Beddoe provides a Standard for Comparison in the Future.*

There was thus a suspicion that city life was shortening stature and favouring the lighter rather than the darker haired types. Unfortunately there had been no Dr. Beddoe in Bristol—nor in any other city—to take a stature and colouring census a century earlier. Dr. Beddoe regretted this loss; he had no past standard with which to compare his findings, but he had the satisfaction of knowing he had provided a standard for the use of those who were to come a century after he was dead and gone—but not forgotten. A man must have public spirit of the highest kind who provides for the needs of those whom he can never know. A nation—a race—can continue to prosper only if they have men and women who build for the future—the type represented by Beddoe.

*A Great Experiment.*

The assemblage of great masses of people in cities is, as I have said, the great anthropological experiment of modern times. Beddoe was the first to take steps to ascertain how city life affected racial types. We look upon cities as the melting-pots of races; we expect Bristolians to epitomize the characteristics of surrounding peoples; we expect the type to become uniform under the influence of city life. Dr. Beddoe found that these expectations were fulfilled in Bristol —up to a certain point. Far from passing towards complete uniformity, however, he found a tendency towards segregation of types; the racial traits which prevailed amongst the business men were not those which marked the artizans; particular crafts seemed to draw into their orbits men of certain frames of body and of aptitude of hand.

*Dr. Beddoe found that Gentlemen Prefer Blondes.*

City life brought about the mingling of races, but it also tended to produce segregation. He accepted evolution as a principle, but never with that depth of conviction which has revolutionized the outlook of modern anthropologists; yet he was convinced that the law of evolution was at work in Bristol. Darwin's law of selection was operative. His figures showed that in selection of their mates the men of Bristol preferred blondes to "reds" and blacks. Disease and conditions of industrial life, on the other hand, favoured those who had a dark complexion rather than those who were fair-skinned. Emigration was selective; it tended to rob Bristol of its best. He perceived that the effects produced by city life on the body and mind of man presented problems of the most complex kind, problems which could be solved only by patient and careful inquiry.

*Evolution is at work in Cities.*

It is this side of Dr. Beddoe's pioneer labours I would press upon your consideration to-day with all my might; we cannot be comfortable concerning our future until we know what is happening to us. The one thing we are certain of is that a city population cannot stand still; evolution is working out our destiny —slowly, insidiously, requiring the passage of centuries to manifest its effects. What I plead for is knowledge of what is happening—the accumulation of such facts as Dr. Beddoe gathered in his time. The medical officer keeps his finger on the pulse of public health; there should be an anthropologist to keep his finger on the pulse of type—physical and mental. There should be a Chair of Physical Anthropology in the University of Bristol.

*Colour as a Key to Race.*

I have said that Dr. Beddoe devoted the spare time of his manhood to amassing data concerning the colouring and other physical traits of the peoples of Western Europe—particularly of communities of the British Isles. What was the net result of all this labour? What was the gain to knowledge? When I

tell you that the gain was two coloured anthropological maps—one of the British Isles, another of Europe, you may feel that Dr. Beddoe's " unequalled " perseverance had a meagre reward. That is not my opinion. I look on these two maps, in which are embodied a lifetime of labour, as the most valuable contribution ever made to our knowledge of the history of the inhabitants of Western Europe. You will find these two maps in his Huxley Lecture—given in 1905—when he was in his seventy-ninth year.[3] The map of Western Europe shows that the dark-skinned people are centred on the Mediterranean, the fair-skinned on the Baltic and North Sea. If we journey across Europe from south to north, we begin in the Mediterranean with people who stand 140 points downwards in Beddoe's scale of nigrescence and end in the Baltic amongst people who rise 150 points upwards in his scale of blondness. Between these two extremes there are peoples showing every intermediate degree of colouring. The transition —the process of blanching—takes place gradually as we pass from south to north; the great neutral zone —betwixt dark and blonde—crosses from east to west, from Switzerland to Belgium and Normandy.

*Evolution of the Peoples of Europe.*

Now if we credit tradition and history, the peoples of Europe in ancient times were in a continual state of unrest, marching hither and thither; there ought not to be this approximate uniformity in the distribution of colour—the most reliable index of race known to us. What is the explanation of this distribution of colour—not Dr. Beddoe's explanation— but the one now offered by modern anthropologists ?

There is only one explanation, namely, that evolution is true, and that in spite of raid and counter-raid in past times, blondness has progressively prospered in Northern Europe, while nigrescence has held its own, or nearly held its own, in Southern Europe. If you ask me why the opposite did not happen—why blondness did not appear in the Mediterranean and nigrescence persist around the Baltic, I have to own that our ignorance, at present, is such that we can give no definite answer. We have to take facts as we find them; more than anyone Dr. Beddoe supplied us with the facts.

*Britain's Racial Frontier.*

When we examine Dr. Beddoe's map of the British Isles we see that the continental frontier zone, between the fair and dark, when it reaches the English Channel, bends suddenly northwards, passing upwards between England and Wales to ultimately emerge at Caithness. Why should our British frontier line lie thus? For an explanation you have to realize that sea power is an ancient factor in the spread of races. The North Sea and English Channel lie, and always have lain, in blonde territory; the Irish Sea, and our western coasts from Land's End to Cape Wrath are now, and were in ancient times, extensions of the Bay of Biscay and of the Mediterranean, highways from the darker zone of Europe to Britain.

*The Old and the New Way of Reading Racial History.*

Now I know very well that if Dr. Beddoe had been with us to-day and occupied a seat on this platform my explanation would have made him turn uneasily in his chair. He was born in a period when men believed that all that can really be known about the history of our ancestors was just what ancient writers have told us about them. The strange fact is that Dr. Beddoe was the first to show us a better way; that our racial history is written in our hair, our eyes, our skin, our skulls, our faces, our bodies and our temperaments. His life-long struggle was to reconcile the new knowledge with the old—not the old with the new—which is our modern aim. He accepted the traditional belief that there was only one gateway to Britain—the Straits of Dover—and that every one of the races which had invaded Britain entered by this portal, sweeping westwards over the racial wave which had preceded it. And yet he observed that the Welsh often reproduced the same type of face as occurs among the Basques of the Western Pyrenees, and that there was a kinship between the people of Spain and of Ireland. If we have shed some of Dr. Beddoe's prejudices, I have no doubt our successors will find that we too have "beams" in our eyes.

*Anthropology and Statesmanship.*

If Dr. Beddoe did seek to make his anthropological observations harmonize with traditional beliefs concerning the origin of British races, yet he never permitted his political outlook to influence his anthropological investigations. He had seen anthropology prostituted in the service of politics. In the sixties of last century two issues were hotly debated: Was the negro of the same species as the white man, or did he represent a lower and separate species? Were the people of Ireland a separate race of mankind, or was the claim of race a mere pretence—

as Huxley thought—for political separation ? Beddoe saw that, when anthropology was yoked to politics, it lost all claims to being a science. He was convinced that politics and anthropology had nought to do with each other. That was not Huxley's belief, nor is it mine. Anthropology stands to statesmanship just as economics does. It is not the business of the economist to determine the policy of his country, but it is his duty to supply statesmen with the data on which sound policy must be framed. It is the business of anthropology to supply the voting public with an ample supply of well-ascertained facts concerning human races and the significance of racial claims. Anthropology should never have been permitted to divorce itself from the living issues of the modern world. It is the duty of the anthropologist to understand human nature and to ascertain how far it must be humoured and how far it can be moulded to fit the needs of an ever-changing civilization.

*We treat the Fact–gatherer unjustly.*

I have said it is the business of anthropology to gather from every available source facts which throw light on the origin and nature of nationalities and races, for in our modern view every nationality is a potential or incipient race. Now Dr. Beddoe performed this service for Britain far beyond any man of his time, or of any time. I ask you to note how we treat men who render our country such a signal service. In 1885 Dr. Beddoe published his greatest book, *The Races of Britain.* If you consult it you will find it contains summaries of observations made in 200 districts of England, 27 in Wales, 102 in Scotland, and 71 in

Ireland. To these he added observations made in 47 parts of France, 9 in Holland, 26 in Belgium, 39 in Germany, 23 in Austria, 22 in Italy, and 11 in Asia Minor. Into this book he compressed thirty-two years of devoted labour. He had to appeal to his friend Arrowsmith in Bristol to publish the book. What was its reception ? It passed through the press when its author was in Australia. "On my return to England," he writes in his Autobiography, "I found my *Races of Britain* on the market. It has never sold so largely as my worthy publisher and I had hoped, but it remains as yet the standard authority on the subject. . . . It is much quoted, and copies once obtained seem to be held somewhat tenaciously, for it never occurs in second-hand catalogues. My friend Topinard, in reviewing it, said I had followed out my subject with a perseverance of which there was no other example." This crumb of comfort from his French friend Topinard made Dr. Beddoe content. No one proclaimed then what the book was and is : the greatest treasury of anthropological fact which has ever appeared in any language.

*How amends can best be made to Dr. Beddoe.*

We were unjust to Dr. John Beddoe, the worthy physician of Bristol, and it is time we made the best amends possible. We are unfair to our "patient gatherers of fact" ; we belaud the architects of specious theories and are apt to treat the makers of our scientific bricks with contumely. Orthodox honours came John Beddoe's way ; he was elected to the Royal Society in 1873, became an LL.D. of Edinburgh in 1885. and elected President of the

Anthropological Institute of Great Britain and Ireland in 1889. Such honours were not commensurate with his deserts. I think it is high time we did something to acknowledge his great and devoted service to his country. In what way can we honour his memory better than by seeking for the means to establish in this University a Chair of Anthropology? You honoured Dr. John Beddoe when this University came into existence by electing him your honorary Professor of Anthropology. It is a real chair we want now—a chair which will permit its occupant to teach, to research, and to bring up a family. I know the times are not favourable for such an appeal, but I have observed that it is just in times of domestic financial stress that men and women permit their generous feelings to run away with them. I hope such a spirit may appear among us now.

*Anthropology as it was and as it must be in the Future.*

I want you to observe that the latter years in Dr. Beddoe's life covered an important period in the history of British anthropology. He was the great amateur in his chosen subject; he was a volunteer anthropologist. Throughout the nineteenth century anthropology was dependent on the amateur or volunteer for its advancement. In every branch of knowledge there comes a time when the professional becomes necessary; to master his subject and to advance it a man must give to it his undivided energies. Anthropology reached this stage in its evolution with the present century; professionalism became imperative. Now universities are just as necessary for professionalism in anthropology as clubs are for professionalism in sport—in cricket, in golf and in football. In order that a professional golfer may exist a club must provide him with a billet; in return he renders tuitional services to the club. In order that a professional anthropologist may exist and develop his subject to the highest point possible a university must provide him with a chair; in return he gives a service of knowledge to the university and to the community in which that university is situated. Think for a moment what the present state of anthropology might have been if Bristol could have offered young Beddoe a chair which permitted him to devote his undivided energies to his chosen subject! I say to you: Better late than never! Institute the chair now. You have your "brain belt" near by. Bristol can depend on a supply of Beddoes. For your own sakes, for the future of England, you should provide for them.

*The force of Dr. Beddoe's example.*

The force of Dr. Beddoe's example is not spent. Twenty-five years ago the lecturer on geography in the University College of Aberystwyth, Professor H. J. Fleure, improved Beddoe's methods and began to apply them to local communities in Wales. One has but to study his results—published after eighteen years of voluntary labour, to perceive how manifold are the types which go to make up a people we are apt to regard as uniform. Professor Fleure found, just as Dr. Beddoe did, that in many country districts there are "pockets" of people, people of a uniform type. In such pockets we see evolution at work—striving to produce a local type or race. Even more important, from my point of view, are the investigations now being carried out by Miss Flemming, a disciple of Professor Fleure. She is following the growth of Welsh children—growth of body, growth of head, changes in colouring—from early childhood to late adolescence. She is finding that every child has its own pattern of growth, just as it has its own feature of face. She is noting the order in which racial characteristics become manifest. Such inquiries are likely to throw light on the evolution of new racial types. I mention these inquiries to remind you that Dr. Beddoe's teaching and example are not dead, and that there awaits the occupant of the proposed Chair of Anthropology in the University of Bristol an infinite number of similar problems which need solving.

*Bristol offers unrivalled opportunities for Anthropological Research.*

From 1810, when Dr. Prichard began practice, until Dr. Beddoe's death in 1911, for a full century, Bristol was known throughout the learned capitals of Europe as a centre for the study of human races. I should be doing a grave injustice were I to omit to say that this university is now the home of a strong school of anthropology. The Spelæological Society, which has its home in Bristol University, is one of the most enterprising voluntary schools to be found anywhere in Europe. Its researches in the caves of the Mendips have added a new chapter to our knowledge of England and of her people in very ancient times. If there be any here who complain of the hardships of our modern way of living, I would beg them to study the *Proceedings* of the Spelæological

Society, and compare the privileges, the opulence, at our disposal compared with what the old cave-dwellers had to put up with. Or if they would know how our ancestors fared in late Celtic times, let them study the revelations made by the archæologists at Glastonbury and at Meare. Why, Bristol is ripe —more than ripe—for the institution of a Faculty of Anthropology. No university, no city, no locality can rival Bristol in the opportunities she offers for the scientific study of mankind.

*A Chair in Memory of Dr. John Beddoe.*

Above all, I would plead for the institution of a Chair of Anthropology in this university as an act of justice due to the memory of a great citizen of Bristol—the late Dr. John Beddoe. I only wish he were here to know that his unselfish labours are not forgotten, and that the city and university with which he was so closely associated now desire to do him honour and to perpetuate his memory as an example for future generations.

REFERENCES.

[1] *Memories of Eighty Years*, 1910.

[2] *English Men of Science*, 1874.

[3] "Colour and Race," *Journ. Royal Anthrop. Institute*, 1905, viii. 219.

